# A 36-Hour Unplugged Full-Scale Exercise: Closing the Gaps in Interagency Collaboration between the Disaster Medical Assistance Team and Urban Search and Rescue Team in Disaster Preparedness in Taiwan

**DOI:** 10.1155/2021/5571009

**Published:** 2021-04-06

**Authors:** Ning-Ping Foo, Edmund Cheung So, Nai-Chen Lu, Shih-Wei Hsieh, Shih-Tien Pan, Yu-Long Chen, Yu-Cheng Hung, Siu-Fung Wong, Chi-Feng Hsu, Chung-Yu Chen

**Affiliations:** ^1^Department of Emergency Medicine, An Nan Hospital, China Medical University, Tainan, Taiwan; ^2^Graduate Institute of Medical Sciences, Chang Jung Christian University, Tainan, Taiwan; ^3^Department of Anesthesia, An Nan Hospital, China Medical University, Tainan, Taiwan; ^4^Department of Nursing, An Nan Hospital, China Medical University, Tainan, Taiwan; ^5^Department of Emergency Medicine, Chi-Mei Medical Center, Liouying, Tainan, Taiwan; ^6^Department of Emergency Medicine, Taipei Tzu Chi Hospital, Buddhist Tzu Chi Medical Foundation, Taipei, Taiwan; ^7^Department of Emergency Medicine, Chang Gung Memorial Hospital, Linkou, Touyuan, Taiwan; ^8^International Association of Emergency Manager, Hong Kong Special Administrative Region, Hong Kong, China; ^9^Department of Emergency Medicine, Ditmansion Medical Foundation, Chia-Yi Christian Hospital, Chiayi, Taiwan; ^10^Department of Occupational Safety and Health, School of Safety and Health Sciences, Chang Jung Christian University, Tainan, Taiwan; ^11^Occupation Environment and Food Safety Research Center, Chan Jung Christian University, Tainan, Taiwan

## Abstract

**Introduction:**

Disaster medical assistance team (DMAT) and urban search and rescue team (USAR) need to cooperate seamlessly to save lives in disasters, but related research is limited.

**Objectives:**

To estimate the disaster preparedness of the DMAT and the barriers affecting interagency cooperation between the DMAT and the USAR team.

**Methods:**

This was an observational study of a full-scale exercise conducted in Taiwan from November 16 to 18, 2018. The exercise scenario simulated a magnitude 7 earthquake in Tainan City. DMATs from other counties were deployed and cooperated with local USAR teams to carry out disaster relief. Our study invited 7 experts to evaluate DMATs on disaster preparedness capabilities and the interagency collaboration between DMATs and USAR.

**Results:**

A total of eight DMATs, consisting of 30 physicians, 65 nurses, 74 logisticians, 5 health bureau personnel, and 85 USAR teams, participated in this exercise. During the mission, 176 patients were treated. The capabilities of each team were generally consistent with the basic technical standards for type I emergency medical teams, but the compliance rates for basic local anesthesia, cold chain equipment for medication, rapid blood test tools, and sterilization devices were only 50%, 12.5%, 12.5%, and 9%, respectively. In addition, 53% of participants reported abnormal vital signs, indicating that it was a high-stress situation. Moreover, the main barriers to interagency collaboration were differing perspectives and poor mutual understanding.

**Conclusion:**

A full-scale exercise carried out jointly with DMATs and USAR teams was valuable for disaster preparedness, particularly in terms of understanding the weaknesses of those teams and the barriers to interagency collaboration.

## 1. Introduction

The evolution of human beings is a history of painful striving in the face of disasters. Many people think that disasters are rare, but there were approximately 262 disasters every year during 2006–2015 [1, 2]. Of the major natural disasters that have occurred worldwide over the past two decades, the most lethal disaster was undoubtedly the Sumatra, Indonesia, tsunami in 2004, with 283,000 deaths [3], followed by the 7.0 magnitude earthquake in Haiti in 2010, with approximately 222,500 deaths [4], and Cyclone Nargis in Myanmar in 2008, with approximately 138,000 fatalities [5]. Taiwan is an island surrounded by the ocean and located at the junction of the Eurasian Plate and the Philippine Sea Plate. Natural disasters, such as earthquakes and typhoons, occur very frequently. The three deadliest natural disasters that have occurred in the past two decades were magnitude 7.3 Chi-Chi earthquake in 1999, which took 2,415 lives [6, 7]; Typhoon Morakot in 2009, which killed 702 [8]; and the magnitude 6.4 Tainan earthquake in 2016, in which 117 died [9].

After each of these disasters, medical volunteers from all over the world helped the regional government perform patient triage, provided high-quality medical care in adverse and spartan environments, or augmented overloaded local health systems. At the national level, for example, in the United States, Japan, and Taiwan, these teams are called disaster medical assistance teams (DMATs) [10–12]. At the international humanitarian mission level, there are various teams that play different roles in the disaster field, for example, the Emergency Medical Team (EMT) of the World Health Organization, the International Committee of the Red Cross, and nongovernment organizations such as Médecins Sans Frontières.

DMATs are roughly divided into three categories: local, national, and international. After the Chi-Chi earthquake, Taiwan set up two national DMATs and other local DMATs [13]. These teams receive regular training and education in disaster preparedness. In the event of a disaster, DMAT members work in safe areas, whereas urban search and rescue (USAR) teams work in hazardous areas. A member of a USAR team is a professional who specializes in rescue work in collapsed buildings, landslide sites, or cars trapped in major traffic accidents. Their tasks are the location, extrication, and initial medical stabilization of trapped victims. Research on DMATs and USAR teams participating together in the same exercise has been limited. Therefore, An Nan Hospital of China Medical University and the Fire Bureau of Tainan City held a 36-hour unplugged, full-scale exercise involving both entities to test their disaster preparedness and interagency collaboration. The reason that the two institutions jointly organized the exercise was mainly because they signed a memorandum of cooperation on February 5, 2018.

## 2. Materials and Methods

### 2.1. Scenario and Settings

This was an observational study without intervention. The exercise was conducted by An Nan Hospital of China Medical University and the fire bureau of Tainan City from November 16 to 18, 2018. The exercise scenario simulated the occurrence of a shallow 7.0 magnitude earthquake in Tainan City that caused the collapse of buildings, power outages, and casualties in multiple districts. The city government immediately established a Local Emergency Management Agency (LEMA) to cope with the disaster. The remote district of Xinhua in Tainan City had heavy casualties during the earthquake. In addition to collapsed buildings (scene A), it also had serious traffic accident events (Scene A), and landslides occurred in a nearby village (Scene B). The local USAR team had been sent to these areas on rescue missions, but local medical supplies were limited. Therefore, DMATs from other counties were activated and deployed in Xinhua District. The exercise lasted from 1800 hours on November 16, 2018, to 0600 hours on November 18, 2018, a total of 36 hours. The LEMA assigned the Ministry of Health to set up a DMAT coordination cell (DMAT-CC) on the spot and received support from the DMATs dispatched by other counties and cities to set up two temporary medical stations to assist the victims at Scene A and Scene B. The distance between Scene A and Scene B was approximately 6 kilometers.

### 2.2. Command, Communication, and Coordination System

**Figure 1:**
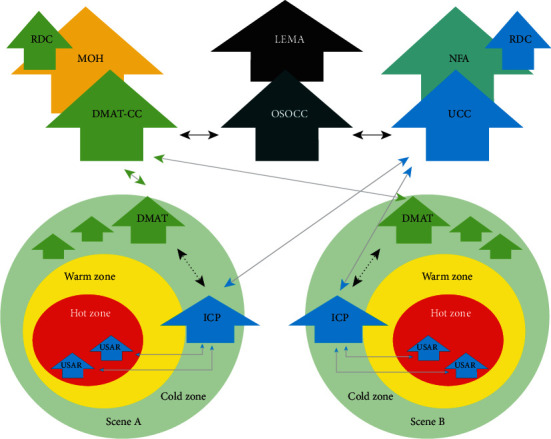
The command and coordination system of this exercise. RDC: Reception and Departure Center; MOH: Ministry of Health; DMAT: disaster medical assistance team; DMAT-CC: DMAT coordination cell; USAR: urban search and rescue; UCC: USAR coordination cell; LEMA: local emergency management agency; NFA: National Fire Agency; OSOCC: on-site operation coordination cell; ICP: incident command post.

The command and coordination system of the exercise is shown in Figure 1. In this figure, there are two disaster areas, Scenes A and B. Each area is divided into a hot zone, a warm zone, and a cold zone. The DMATs worked either individually or in collaboration to set up a medical station in the cold zone, and they were guided by the DMAT-CC. The USAR teams worked together in the hot zone and formed an incident command post (ICP) at each disaster scene, and their commander was the USAR coordination cell (UCC). The on-site operation coordination cell (OSOCC) was the agency assigned by the LEMA to coordinate among the different agencies, and it had regular meetings with the DMAT-CC and UCC to formulate disaster relief policies and determine disaster relief goals. In this exercise, the coordinators of the DMAT-CC were alternately played by 5 members of the Ministry of Health from three counties and cities. In addition, an incident radio communication plan (ICS 205) was set up before the exercise, with at least 9 radio channels for different zones and agencies.

### 2.3. Participants, Objectives of the Exercise, and Assessment Criteria

The DMATs invited to participate in this exercise needed to be self-sufficient, including with respect to their electrical supply during the entire mission. There were two objectives of this exercise. The first objective was to determine whether the DMAT met the basic technical standards during the mission. The assessment criteria for the exercise were based on the minimum technical standards of self-assessment for type I Fixed Emergency Medical Teams set forth in the EMT coordination handbook [13]. These skills included initial assessment and triage, resuscitation, patient stabilization and referral, wound care, fracture management, anesthesia, and surgery. The second objective was to identify barriers to cooperation between the DMATs and USAR teams. The organizer invited six experts from Taiwan's Society of Emergency Medicine and a nongovernmental expert from Hong Kong well trained in humanitarian relief missions to serve as exercise controllers and evaluators. Each evaluator was responsible for assessments at different times and in different disaster scene areas. They participated in regular meetings with the DMATs and USAR teams in the OSOCC every morning; monitored all teams, including all radio channels used for communication between the DMAT-CC and the UCC; and visited the disaster area to observe the disaster relief and rescue operations in action to assess whether there were any barriers to cooperation between the different teams. On the last day of the exercise, all evaluators met and conducted internal discussions prior to issuing a formal report.

To minimize the cost of exercise, the members of the DMATs served as mock patients outside of duty hours to test the other DMATs. Mock patients were realistically made up to create a sense of authenticity. During the exercise, all medical actions, including medical recordkeeping, physical examination, wound dressing or suture, plaster fixation, medication prescription, and discharge arrangements, needed to be carried out accurately. In addition, if the patients were to be transferred to the hospital, they would have to be moved to an ambulance and sent away for the task to be completed. The ambulances were provided by the Fire Bureau of Tainan City.

### 2.4. Health Assessment of Team Members

To ensure the safety of the participants, we required each participant to upload his or her vital signs, including blood pressure, heart rate, respiratory rate, body temperature, room-air pulse oximetry, and any symptoms of physical or mental discomfort, to the online database at least once a day. In this way, the medical staff on each team and the organizer could closely monitor the team members to ensure that everything was in good order.

### 2.5. Statistical Analysis

All data were analyzed using Microsoft Excel 2018 software. Descriptive analysis was used in this study in which categorical variables were presented as numbers and percentages, and continuous variables were presented as the mean and standard deviation.

## 3. Results

### 3.1. Participants

Eight disaster medical rescue teams participated in this exercise. A total of 30 physicians, 65 nurses, 74 logistics staff (paramedics and administrative staff), 5 health bureau staff, 85 USAR members, and 7 controllers were involved. The farthest team came from 473 kilometers away, and the nearest team was only 16 kilometers away from the disaster scene.

**Figure 2:**
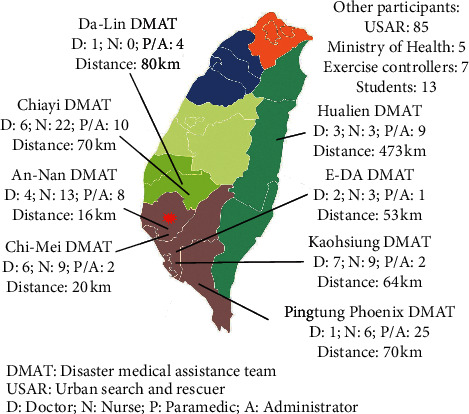
The basic characteristics of DMATs in this exercise.

Among the members of the DMATs, there were 97 males (57.4%) and 72 females (42.6%). The average age was 34.4 ± 8.3 years, with a range of 20–60 years. Among the participating physicians, there were 27 emergency physicians, 1 anesthesiologist, 1 pediatric surgeon, and 1 orthopedist. The other information is listed in Figure 2.

### 3.2. Characteristics of the Simulated Patients

A total of 176 injured patients were treated during exercise, of which 18 (10.2%) were younger than 17 years old, 9 (5.1%) were older than 65 years old, and 147 were trauma patients (83.5%). Among them, 116 patients (65.9%) were discharged from the medical station posttreatment, but 56 patients (31.8%) were referred to the hospital by ambulance for further treatment. Two of them (1.1%) died at the medical station. They did not receive first aid but were declared dead directly by the physician. Three patients (1.7%) required amputation on the spot; one amputation was performed by doctors on the USAR medical team, and two were performed by DMAT doctors. Eleven patients (6.2%) with acute gastroenteritis were reported to have DMAT-CC on the morning of the third day, and the DMAT-CC was alerted to the possibility of cluster infection in a timely manner. The other information is listed in Table 1.

### 3.3. Technical Standard Evaluation for the DMATs

The overall compliance rate with the technical standards for type 1 fixed EMTs was 70.4% (Table 2). The compliance rate was good for most of the assessment standards, but several standards were not met. All DMATs had devices for primary wound closure, but half of them neglected to bring local anesthesia. Only one DMAT (12.5%) had cold chain equipment for tetanus toxoid storage, and one DMAT (12.5%) had the capacity to perform basic rapid detection tests by using i-STAT. None of the DMATs were capable of performing basic steam autoclaving or had enough disposable medical equipment, such as surgical devices or intubation devices. Two of the teams (25%) had portable ultrasound devices that could be widely used in the disaster field.

### 3.4. Health Assessment

During the exercise, a total of 444 data points were collected, of which 12 data points were collected on the first day, 343 on the second day, and 89 on the third day. Each participant on each team entered at least one set of data. The participants in the exercise were all healthy, but the monitoring results showed that there were 235 (52.9%) abnormal results, including 137 (30.9%) instances of elevated systolic blood pressure > 130 mmHg.

A total of 186 (41.9%) patients had elevated diastolic blood pressure > 80 mmHg, and 46 (10.4%) reported relative tachycardia with a heartbeat > 100 beats/minute. In addition, two of the team members complained of insomnia, one complained of stress-induced headache, and another quit the mission due to acute gastroenteritis. The remainder of the information can be seen in Table 3.

### 3.5. Command, Control, and Communication

**Figure 3:**
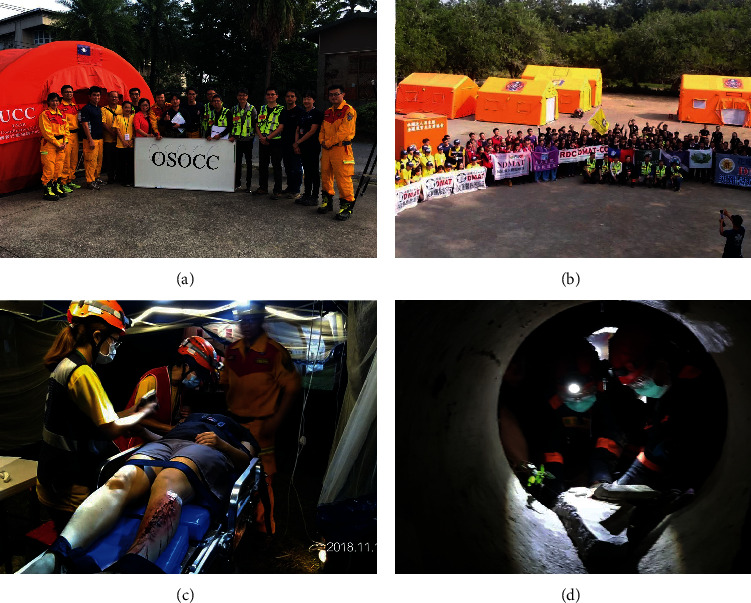
Photographs of the full-scale exercise. (a) Group photo of the core leader of each unit after the meeting at OSOCC; (b) group photo of DMATs in the base of operation; (c) part of the medical station in Scene B; (d) DMAT doctors performing emergency amputation surgery in a confined space.

According to the exercise evaluators, the DMAT-CC was effective in many respects, including leadership, coordination, and quality assurance, and provided excellent support services for each team. The communication by radio went smoothly after the organizer provided guidance for communication professionals. In addition, the study found an interagency barrier between the DMAT and USAR teams, leading to delays during rescue missions. These barriers were related to differing perspectives on safety zones during disasters and poor mutual understanding of each other's capabilities. Detailed information is described in the discussion section. Furthermore, Figure 3 presents photographs to more clearly depict the exercise scenarios.

## 4. Discussion

Although natural disasters are perceived as inevitable events that lead to tremendous casualties, their negative impact can be diminished through the preparedness of the population. The Federal Emergency Management Agency divides disaster management into four phases: mitigation, preparedness, response, and recovery. Among these, full-scale exercise is an important tool to evaluate disaster preparedness. There are at least two advantages in conducting an exercise. First, the strengths and weaknesses of disaster preparedness can be detected so that these weaknesses can be improved in the follow-up plan. For example, Lee et al. [14] reported their experience with the Korea Disaster Relief Team in joining the ASEAN regional forum disaster relief exercise during 2013. This survey showed insufficient disaster preparedness, particularly in the need for improved communication and the proper management of awareness and operation of the incident command system. Second, the experience that the exercise brings to the participants can effectively improve personal abilities and strengthen psychological resilience. For instance, in a report of terror attacks in Norway, 2011, it was found that rescue personnel who had more years of experience, previous training and/or drills before, and prior mass casualty experiences had a lower prevalence of posttraumatic stress disorder and a higher level of understanding of personal role clarity [15].

There is no uniform standard to be tested in disaster exercise, and adjustments are made according to the objectives of the exercise. Albarqouni et al. [16] in Australia used a consensus statement based on systemic review and a Delphi survey to determine the core competencies of health professionals. Yoon and Choi [17], in Korea, divided the core competencies of disaster mental health workforce members into individual competences and organization competences. As an organizer of the exercise, he or she can use these competencies to test the achievement percentages as an outcome of the exercise. However, in this 36-hour full-scale exercise, the competences to be tested are part of the compliance rate of WHO EMT Type I fixed technical standards. The main reason is that it is a standard published by the WHO, which is more suitable for the role of DMAT in disaster preparedness and deployment.

The compliance rates of initial assessment and triage, resuscitation, patient stabilization and referral, wound care, fracture management, surgery (minor outpatient procedure), and emergency care for chronic disease in the survey were 100%. The possible reason was that DMAT teams consisted of doctors who were experienced emergency physicians, the majority of the nurses were emergency nurses or had received disaster training nurses, and the majority of the logisticians were paramedics that owned emergency medical technician licenses who were familiar with resuscitation and first aid. During disaster relief, it is very important to have trained nurses participating in the mission. Referring to other countries, such as Poland, Goniewicz et al. [18] reported a disaster preparedness survey on nurses from all medical centers in Lublin and found that only 16.1% of nurses had accepted triage training, 4.1% had HAZMAT training, and 11.3% had advanced cardiac life support training. This shows that if a disaster truly occurs, it is questionable whether these nurses have the ability to handle rescue missions. In contrast, if it is through a complete training course, these problems can be improved. According to the standard course for burn disasters in Kansas, health care providers can significantly improve the abilities, confidence, and competence of burn assessments and treatment modalities through training [19].

This study detected weaknesses with respect to compliance with technical standards, including the preparation of local anesthesia, the lack of laboratory test tools, cold chain equipment for tetanus toxoid storage, and sterilization devices. We believe these problems can be overcome in subsequent preparations, since the lack of compliance was due mainly to a lack of funds and not to insufficient training. On the other hand, two DMATs brought portable ultrasound devices to the disaster field, and they used them to scan patients with suspected tension pneumothorax, internal bleeding, pericardial effusion, and long bone fractures and to evaluate patients' hemodynamic status, which was very helpful in the disaster scenario. Shorter and Macias [20] reported the use of portable ultrasound devices after the Haiti earthquake, which influenced decisions on patient care in 70% of scans. Wydo et al. [21] also observed that the use of ultrasound can help in the rapid evaluation, triage, and treatment of injured patients in disasters. Based on previous studies and the experience of this exercise, ultrasound is strongly recommended for use in mass casualty incidents and disaster triage, even though it is not the standard equipment in the self-assessment minimum technical standards for type I EMTs.

Nurthen and Jung [22] reported on fatalities in the Peace Corps during 1986–2003; the major cause of death was unintentional injury (67%), and half of these injuries were caused by motor vehicle accidents. Another study of health problems in a group of volunteers or humanitarian aid workers in post-earthquake Nepal showed that 53 (56%) had gastrointestinal illness, 14 (15%) had skin diseases, and 7 (8%) had injury/musculoskeletal problems [23]. However, these issues were rarely mentioned in the full-scale exercise. Our study found that 52.9% of recorded vital signs were abnormal, with numerous cases of high blood pressure and relative tachycardia, even among younger personnel. Many participants stated that, in this highly realistic exercise, they were working under high pressure, and most of them rested less than 6 hours during the 36-hour exercise, which may explain their unstable vital signs. Members participating in the exercise must understand that the disaster response involves serious physical and psychological pressure, but the organizer must also achieve a balance between high fidelity and the development of burnout or posttraumatic stress syndrome.

Disaster events often involve multiple professions and agencies, and different professions or agencies may have different terms for and understandings of responsibilities, facilities, personnel identities, and equipment. This makes coordination and communication between them difficult, resulting in an inability to integrate operations and low efficiency. The exercise demonstrated two major interagency barriers between the USAR and DMAT: differing perspectives and poor mutual understanding. Here, we present some examples of these barriers. First, in the exercise scenario, a tour bus was involved in a traffic accident during the earthquake. USAR personnel rescued many injured patients. When they asked the DMAT for medical treatment assistance at the scene, the request was rejected by the DMAT-CC, causing injured patients to stay at the crash site for more than 1 hour. The two coordinators had a dispute on the radio channel. After a long coordination period, it was found that the DMAT-CC believed that the disaster scene was a red zone and entry was not allowed, while the USAR team thought the area was a cold zone.

The second example occurred late at night the following day. USAR personnel attempted to free an injured patient trapped in a collapsed building. The patient's right forearm was crushed by a heavy object, and his vital signs were unstable. He needed an emergency amputation procedure to save his life. The USAR medic did not have the equipment to perform the amputation, and therefore, the UCC requested the DMAT-CC to assign a team to perform the operation, but the request was again initially rejected by the DMAT-CC for the same reason. After nearly 1.5 hours of coordination, the DMAT-CC assigned doctors and nurses who had been trained to work in confined spaces, and they completed the amputation operation. Both of these issues were raised at the regular OSOCC meeting the next day. Participants discussed their differences with open minds to reach understanding and consensus. Interestingly, the USAR personnel believed that all DMAT doctors can perform amputations in a confined space; they did not know that only a few experienced physicians can.

Schmitz et al. [24] described a collaboration between emergency managers at a local US Department of Veterans Affairs Medical Center and Veterans Affairs entities, and they found that potential partners viewed a Veterans Affairs Medical Center partnership with skepticism. Gesser-Edelsburg et al. [25], in a study titled Gaps in Public Health Emergency Preparedness in Israel, revealed multiple barriers, including obstacles related to interagency coordination and interfacing, interdisciplinary integration, and communication. Cooper et al. [26], in a review of interagency collaboration related to children's and young people's mental health, demonstrated that the most commonly perceived barriers to interagency collaboration were inadequate resourcing, poor interagency communication, lack of valuing across agencies, differing perspectives, poor understandings across agencies, and confidentiality issues. Those studies demonstrated that the barriers to interagency partnerships in different fields are similar. Therefore, to close the gap in interagency collaboration between the DMAT and USAR, particularly in differing perspectives and poor mutual understanding, we recommend the signing of a memorandum of understanding between the agencies, joint training and colocation of staff, resource allocation, and the use of standard operation procedures in the future.

There are many advantages to a long, uninterrupted exercise, including allowing participants to experience the arduous nature of shifts, the difficulty of manpower scheduling, the lack of logistics supplies, and coordination between various agencies. This experience has not been available in past exercises that lasted only a few hours. However, there were still some limitations of this study: the exercise does not have a full comprehensive qualitative and quantitative analysis, such as the correct treatment score of each patient and others. In the original design, this quantitative indicator was scored by simulated patients to evaluate DMAT team members. However, because simulated patients did not have standardized performance and unified evaluation standards, the data were unable to be analyzed. Moreover, the main outcome of this research was an evaluation, and there could have been an effect of subjectivity. However, the results of the evaluation were discussed by all evaluators, which reduced the variability among different evaluators. In addition, regarding the measurement of vital signs, the study found that a large number of participants had abnormal vital signs; however, the researchers did not measure the vital signs of the participants before the exercise, making it impossible to perform a statistical comparison before and after the test. In fact, the participants were all healthy and from the same ethnic groups, and a large number of abnormal values is still an important finding. Other technical standards for a type 1 fixed EMT, such as those regarding outpatient care for communicable diseases, emergency obstetric care, outpatient pediatric care, rehabilitation, and mental health care, were not tested in this exercise. Last, some institutions did not join the exercise, including the police bureau, environmental protection bureau, and others. These units may need to work together at the scene of a disaster. We hope that, in the future, we can invite all relevant institutions to participate in a full-scale exercise.

## 5. Conclusions

This full-scale exercise successfully achieved the preset goals and evaluated the shortcomings in disaster preparation. Cooperation jointly with DMATs and USAR teams was valuable in disaster preparedness, particularly in terms of understanding the weaknesses of those teams and the barriers to interagency collaboration.

## Figures and Tables

**Table 1:** The basic characteristics of the participants in this exercise.

Characteristic	Items	*N* = 176	%
Sex	Male	120	68.2
	Female (not pregnant)	53	30.1
	Female (pregnant)	3	1.7

Trauma	Abrasion/contusion	45	25.6
	Fracture	30	17.0
	Laceration/soft tissue defect	28	159
	Internal bleeding (chest/abdomen)	13	7.4
	Sprain or stretch	12	6.8
	Burn	5	2.8
	Ophthalmic injury	4	2.3
	Amputation	3	1.7
	Other	7	4.0

Non-trauma	Acute gastroenteritis	11	6.2
	Acute upper respiratory disease/asthma	4	2.3
	Hypertension symptoms	3	1.7
	Diabetes mellitus with hyperglycemia	2	1.1
	Allergy syndrome	2	1.1
	Other (including one suspected stroke)	6	3.4

Follow-up	Discharge without follow-up	87	49.4
	Discharge with follow-up	29	16.6
	Referral/transfer	56	31.8
	Left without medical advice	2	1.1
	Dead upon arrival	2	1.1

**Table 2:** The rates of compliance with the basic technical standards for each DMAT (a total of eight DMATs).

Technical standards for type I fixed EMTs	*n*/*N*	Percentage
Initial-assessment and triage	8/8	100.0
Resuscitation	8/8	100.0
Patient stabilization and referral	8/8	100.0
Wound care	8/8	100.0
Fracture management	8/8	100.0
Surgery (minor outpatient procedure)	8/8	100.0
Emergency care for chronic disease	8/8	100.0
Local anesthesia	4/8	50.0
Laboratory test	1/8	12.5
Pharmacy and drug supply (tetanus)	1/8	12.5
Sterilization	0/8	0.0

EMT: emergency medical team.

**Table 3:** Self-reported health assessment information for all DMAT members.

Item	Average ± SD	Range
All vital signs	*n* = 444	
Systolic blood pressure	128.0 ± 7.2 mmHg	84–177
Diastolic blood pressure	80.7 ± 5.3 mmHg	50–117
Heart rate	76.0 ± 0.0/min	48–123
Respiratory rate	19.3 ± 1.1/min	10–24
Body temperature	36.0 ± 0.0°C	34.1–38.1
Pulse oximeter	97.8 ± 1.4%	94–100

Data with abnormal vital signs	*N*	Percentage
Systolic blood pressure >130 mmHg	137	30.9
Diastolic blood pressure >80 mmHg	186	41.9
Heart rate >100 (beats per minute)	46	10.4
Respiratory rate >20 (rate per minute)	4	0.9
Body temperature >38°C	3	0.7
Pulse oximeter <95 (%)	2	0.4

^∗^A total of 444 vital sign data points were collected during the full-scale exercise; SD: standard deviation.

## Data Availability

The data used to support the findings of this study are available from the corresponding author upon request.

## Abbreviations

DMAT: Disaster medical assistance team
USAR: Urban search and rescue team
LEMA: Local emergency management agency
OSOCC: On-site operation coordination cell
DMAT-CC: DMAT coordination cell
UCC: USAR coordination cell
EMT: Emergency medical team.

## References

[1] Guha-Sapir D., Wallemacq-Pascaline-Hoyois P., Below R. (2016). *Annual Disaster Statistical Review 2015: The Numbers and Trends*.

[2] Pettersson T., Wallensteen P. (2015). Armed conflicts, 1946-2014. *Journal of Peace Research*.

[3] Lay T., Kanamori H., Ammon C. J. (2005). The great Sumatra-Andaman earthquake of 26 December 2004. *Science*.

[4] Guha-Sapir D., Vos F., Below R., Ponserre S. (2011). *EM-DAT the International Disaster Database*.

[5] Besset M., Anthony E. J., Dussouillez P., Goichot M. (2017). The impact of Cyclone Nargis on the Ayeyarwady (Irrawaddy) River delta shoreline and nearshore zone (Myanmar): towards degraded delta resilience?. *Comptes Rendus Geoscience*.

[6] Hsu E. B., Ma M., Lin F. Y., VanRooyen M. J., Burkle F. M. (2002). Emergency medical assistance team response following Taiwan Chi-Chi earthquake. *Prehospital and Disaster Medicine*.

[7] Liang N.-J., Shih Y.-T., Shih F.-Y. (2001). Disaster epidemiology and medical response in the Chi-Chi earthquake in Taiwan. *Annals of Emergency Medicine*.

[8] Pan C.-L., Chiu C.-W., Wen J.-C. (2014). Adaptation and promotion of emergency medical service transportation for climate change. *Medicine*.

[9] Pan S.-T., Cheng Y.-Y., Wu C.-L. (2019). Association of injury pattern and entrapment location inside damaged buildings in the 2016 Taiwan earthquake. *Journal of the Formosan Medical Association*.

[10] Mace S. E., Jones J. T., Bern A. I. (2007). An analysis of disaster medical assistance team (DMAT) deployments in the United States. *Prehospital Emergency Care*.

[11] Fuse A., Yokota H. (2010). An analysis of Japan disaster medical assistance team (J-DMAT) deployments in comparison with those of J-DMAT’s counterpart in the United States (US-DMAT). *Journal of Nippon Medical School*.

[12] Lin C.-H., Chang W.-H., Wu C.-L., Pan S.-T., Chi C.-H. (2016). Medical response to 2016 earthquake in Taiwan. *The Lancet*.

[13] World Health Organization (2016). *Emergency Medical Teams, Coordination Hand Book*.

[14] Lee J. I., Lee K. H., Kim O. H. (2016). Evaluation of an international disaster relief team After participation in an ASEAN regional forum disaster relief exercise. *Disaster Medicine and Public Health Preparedness*.

[15] Pedersen M. J., Gjerland A., Rund B. R., Ø E., Skogstad L. (2016). Emergency preparedness and role clarity among rescue workers during the terror attacks in Norway july 22, 2011. *PLoS One*.

[16] Albarqouni L., Hoffmann T., Straus S. (2018). Core competencies in evidence-based practice for health professionals: consensus statement based on a systematic review and Delphi survey. *JAMA Network Open*.

[17] Yoon H.-y., Choi Y.-K. (2019). The development and validation of the perceived competence scale for disaster mental health workforce. *Psychiatry Investigation*.

[18] Goniewicz K., Goniewicz M., Burkle F. M., Khorram-Manesh A. (2021). Cohort research analysis of disaster experience, preparedness, and competency-based training among nurses. *PLoS One*.

[19] Wetta-Hall R., Jost J. C., Jost G., Praheswari Y., Berg-Copas G. M. (2007). Preparing for burn disasters: evaluation of a continuing education training course for pre-hospital and hospital professionals in Kansas. *Journal of Burn Care & Research*.

[20] Shorter M., Macias D. J. (2012). Portable handheld ultrasound in austere environments: use in the Haiti disaster. *Prehospital and Disaster Medicine*.

[21] Wydo S. M., Seamon M. J., Melanson S. W., Thomas P., Bahner D. P., Stawicki S. P. (2016). Portable ultrasound in disaster triage: a focused review. *European Journal of Trauma and Emergency Surgery*.

[22] Nurthen N. M., Jung P. (2008). Fatalities in the peace corps: a retrospective study, 1984 to 2003. *Journal of Travel Medicine*.

[23] Bhandari D., Pandey P. (2018). Health problems while working as a volunteer or humanitarian aid worker in post-earthquake Nepal. *Journal of Nepal Medical Association*.

[24] Schmitz S., Wyte-Lake T., Dobalian A. (2018). Facilitators and barriers to preparedness partnerships: a veterans affairs medical center perspective. *Disaster Medicine and Public Health Preparedness*.

[25] Edelsburg A., Cohen R., Diamant A. (2019). Experts’ views on the gaps in public health emergency preparedness in Israel: a qualitative case study. *Disaster Medicine and Public Health Preparedness*.

[26] Cooper M., Evans Y., Pybis J. (2016). Interagency collaboration in children and young people’s mental health: a systematic review of outcomes, facilitating factors and inhibiting factors. *Child: Care, Health and Development*.

